# Divergent effects of muscarinic receptor subtype gene ablation on murine colon tumorigenesis reveals association of M3R and zinc finger protein 277 expression in colon neoplasia

**DOI:** 10.1186/1476-4598-13-77

**Published:** 2014-04-03

**Authors:** Kunrong Cheng, Guofeng Xie, Sandeep Khurana, Jonathon Heath, Cinthia B Drachenberg, Jennifer Timmons, Nirish Shah, Jean-Pierre Raufman

**Affiliations:** 1 Department of Medicine, Division of Gastroenterology & Hepatology and Program in Oncology, Marlene and Stewart Greenebaum Cancer Center, VA Maryland Health Care System and University of Maryland School of Medicine, Baltimore, MD 21201-1595, USA; 2 Department of Medicine, Division of Gastroenterology, Georgia Regents University, Augusta, GA 30912, USA; 3 Department of Pathology, Division of Anatomic Pathology, University of Maryland School of Medicine, Baltimore, MD 21201-1595, USA; 4 Department of Surgery, University of Maryland School of Medicine, Baltimore, MD 21201-1595, USA; 5 Division of Gastroenterology & Hepatology, 22 S. Greene St., N3W62, Baltimore, MD 21201-1595, USA

**Keywords:** Muscarinic receptors, M3R, M1R, *Chrm1*, *Chrm3*, Mouse models, Colon cancer, Azoxymethane, ZNF277

## Abstract

**Background:**

M3 and M1 subtype muscarinic receptors are co-expressed in normal and neoplastic intestinal epithelial cells. In mice, ablating *Chrm3*, the gene encoding M3R, robustly attenuates intestinal tumor formation. Here we investigated the effects of *Chrm1* gene ablation, alone and in combination with *Chrm3* ablation.

**Methods:**

We used wild-type, *Chrm1*^*-/-*^, *Chrm3*^*-/-*^ and combined *Chrm1*^*-/-*^*/Chrm3*^*-/-*^ knockout (dual knockout) mice. Animals were treated with azoxymethane, an intestine-selective carcinogen. After 20 weeks, colon tumors were counted and analyzed histologically and by immunohistochemical staining. Tumor gene expression was analyzed using microarray and results validated by RT-PCR. Key findings were extended by analyzing gene and protein expression in human colon cancers and adjacent normal colon tissue.

**Results:**

Azoxymethane-treated *Chrm3*^*-/-*^ mice had fewer and smaller colon tumors than wild-type mice. Reductions in colon tumor number and size were not observed in *Chrm1*^*-/-*^ or dual knockout mice. To gain genetic insight into these divergent phenotypes we used an unbiased microarray approach to compare gene expression in tumors from *Chrm3*^*-/-*^ to those in wild-type mice. We detected altered expression of 430 genes, validated by quantitative RT-PCR for the top 14 up- and 14 down-regulated genes. Comparing expression of this 28-gene subset in tumors from wild-type, *Chrm3*^*-/-*^, *Chrm1*^*-/-*^ and dual knockout mice revealed significantly reduced expression of *Zfp277*, encoding zinc finger protein 277, in tissue from M3R-deficient and dual knockout mice, and parallel changes in Zfp277 protein expression. Notably, mRNA and protein for ZNF277, the human analogue of Zfp277, were increased in human colon cancer compared to adjacent normal colon, along with parallel changes in expression of M3R.

**Conclusions:**

Our results identify a novel candidate mouse gene, *Zfp277,* whose expression pattern is compatible with a role in mediating divergent effects of *Chrm3* and *Chrm1* gene ablation on murine intestinal neoplasia. The biological importance of this observation is strengthened by finding increased expression of *ZNF277* in human colon cancer with a parallel increase in M3R expression. The role of zinc finger protein 277 in colon cancer and its relationship to M3R expression and activation are worthy of further investigation.

## Background

Activation of muscarinic receptors and downstream signaling was shown to stimulate proliferation of cells derived from lung [1,2], breast [3,4], prostate [5], colon [6,7], and skin [8] cancers, and muscarinic receptors are frequently over-expressed in these common cancers [9]. Hence, it is highly likely that activation of muscarinic receptor signaling plays a fundamentally important role in neoplastic transformation and progression.

Of five cholinergic muscarinic receptor subtypes, designated M1R – M5R [10], human colon cancer cells express primarily M3R. M3R activation stimulates cell proliferation, survival, migration and invasion [6,11-14] – key hallmarks of neoplasia [15]. Human colon cancer cells also produce and release acetylcholine at concentrations capable of activating M3R and stimulating cell proliferation, identifying the capacity for autocrine and paracrine stimulation of M3R signaling [11]. Jointly, these *in vitro* studies provided strong evidence that M3R expression and signaling are particularly important in the progression of colon neoplasia.

Mice with targeted knockout of genes encoding each of the five muscarinic receptor subtypes (*Chrm1* – *Chrm5*) are useful for investigating their biological functions [16-18]. We showed that ablating *Chrm3*, the gene encoding M3R, attenuates colon neoplasia in mice treated with azoxymethane (AOM), a colon-selective carcinogen [19]. Compared to AOM-treated WT mice, AOM-treated *Chrm3* knockout mice had 40% and 60% reductions in tumor number and size, respectively. Similar results were obtained using *Apc*^*min/+*^ mice, a genetic model of intestinal neoplasia [20]. These findings suggested to us that treatments directed at reducing M3R expression, activation or downstream signaling might be useful to prevent or treat colon neoplasia. Indeed, *Apc*^*min/+*^ mice treated with scopolamine butylbromide, an inhibitor of muscarinic receptor activation, developed fewer intestinal tumors than vehicle-treated control mice [20].

Many cell types co-express muscarinic receptor subtypes [17]. Using *in situ* hybridization, we demonstrated expression of mRNA for both M1R and M3R in murine gastric [21] and colonic [19] epithelial cells. Likewise, human colon cancer cells used to investigate *in vitro* actions of muscarinic receptors and ligands express a mixture of M3R and M1R, with a predominance of M3R [13,22]. Whereas expression of multiple muscarinic receptor subtypes in the same cell type is likely to provide growth and survival advantages, it can also result in complex, unpredictable interactions. This was apparent when we examined the impact of *Chrm1* and *Chrm3* co-expression in gastric chief cells that synthesize and release the pro-enzyme pepsinogen [21]. M3R deficiency did not alter carbamylcholine (carbachol)-induced pepsinogen release, but M1R deficiency resulted in a 25% decrease in pro-enzyme release [21]. Strikingly, in mice deficient in both M1R and M3R, carbachol-induced pepsinogen secretion was totally abolished [21].

These observations motivated us to examine the role of M1R (*Chrm1*) expression in colon neoplasia and to determine whether knocking out both M1R (*Chrm1*) and M3R (*Chrm3*) in the same animal (hereafter called dual KO mice) would more effectively attenuate AOM-induced colon neoplasia than M3R (*Chrm3*) knockout alone. We also took advantage of these murine models to identify genes whose expression levels might be relevant to resulting colon tumor phenotypes.

## Results

### Effects of M1R, M3R and dual knockout on murine colon neoplasia

**Figure 1:**
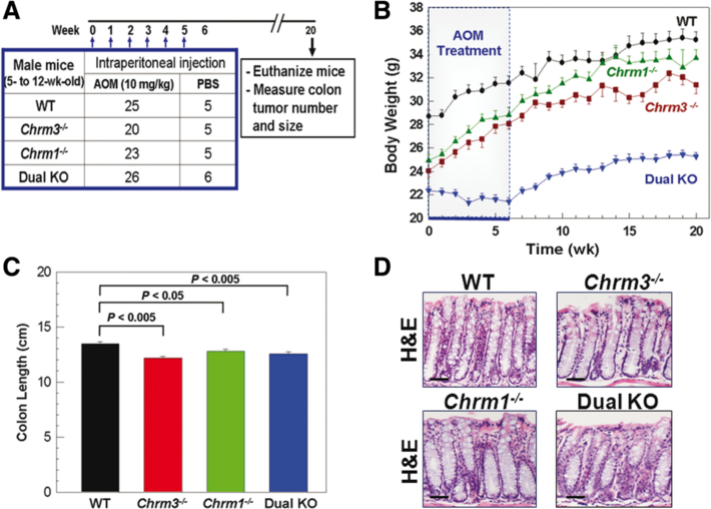
**Study protocol, animal weights, colon length and histological appearance of colon sections from mice with different *Chrm *genotypes. A**: Schematic of study design; WT, *Chrm3*^*-/-*^, *Chrm1*^*-/-*^ and dual KO male mice were treated with intraperitoneal injection of AOM (10 mg/kg) or an equal volume of vehicle (phosphate buffered saline) weekly for 6 weeks and followed for a total of 20 weeks. At 20 weeks, animals were euthanized and colon tumor number and size, and mucosal markers of proliferation and apoptosis were measured. **B**: Weights of AOM-treated mice during the 20-week study (mean ± S.E.). **C**: Colon length of AOM-treated mice was measured following euthanasia at 20 weeks (mean ± S.E.). **D** No morphological differences were seen in hematoxylin and eosin (H&E)-stained microscopic sections of normal colon tissue from WT, *Chrm3*^*-/-*^, *Chrm1*^*-/-*^ and dual KO mice. Size bars = 50 micrometers.

We treated WT, *Chrm3*^*-/-*^, *Chrm1*^*-/-*^ and dual KO mice with phosphate buffered saline (vehicle control) or AOM, a colon-selective carcinogen [19] (Figure 1A). As reported previously [23], at baseline *Chrm3*^*-/-*^ mice weighed 15-20% less than WT mice (Figure 1B). At baseline, *Chrm1*^*-/-*^ mice also weighed less than WT mice, but in contrast to *Chrm3*^*-/-*^ mice, their weights approached those of WT mice by the end of the 20-week study period (Figure 1B).

As a group dual KO mice were extremely frail, necessitating a modified study design; whereas AOM treatment was started in six-week-old *Chrm3*^*-/-*^ and *Chrm1*^*-/-*^ mice, AOM treatment was delayed in dual KO mice until they were 11 to 12-weeks-old and better able to tolerate AOM treatment [24]. Even with this modification, at 11-12 of age dual KO mice weighed ~25% less than WT mice (Figure 1B), and in contrast to the other genotypes dual KO mice lost weight during the first six weeks of AOM treatment; at 20 weeks they still weighed ~30% less than WT mice (Figure 1B).

Although the gross anatomical appearance of colons in knockout mice was normal, we detected a modest but statistically significant reduction in colon length (Figure 1C). These differences probably reflect the lower body weights of *Chrm3*^*-/-*^, *Chrm1*^*-/-*^ and dual KO compared to WT mice (Figure 1B). Colon length was not significantly different when compared within the three groups of knockout mice. Microscopic review of H&E-stained tissue sections from untreated (no AOM or vehicle) mice with the four muscarinic receptor genotypes by a senior gastrointestinal pathologist (CD) revealed no differences in colon epithelial morphology (Figure 1D).

**Figure 2:**
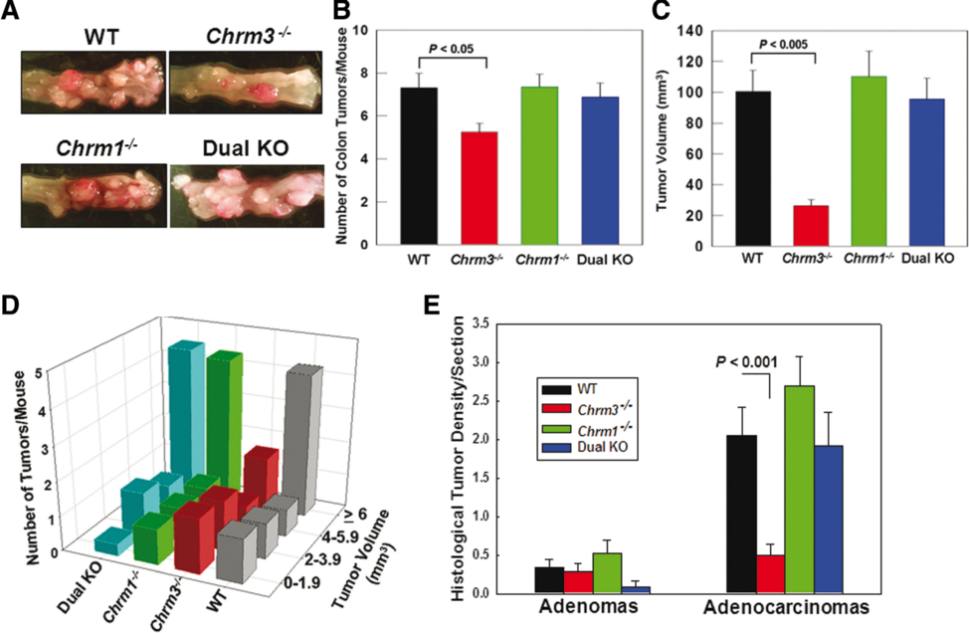
**Measurement of colon tumor number and size 20 weeks after starting AOM treatment. A**: Representative photographs of resected distal colon from AOM-treated mice with different *Chrm* genotypes. **B**: Tumor number was reduced in AOM-treated *Chrm3*^*-/-*^ compared to WT mice (*P* < 0.05). There was no significant difference in tumor number in *Chrm1*^*-/-*^ and dual KO compared to WT mice (mean ± S.E.). **C**: Reduced tumor volume in *Chrm3*^*-/-*^ compared to WT mice (*P* < 0.005). Tumor volume in *Chrm1*^*-/-*^ and dual KO compared to WT mice was not significantly different (mean ± S.E.). **D**: Histogram of tumor volume in AOM-treated WT, *Chrm3*^*-/-*^, *Chrm1*^*-/-*^ and dual KO mice. **E**: Numbers of adenomas and adenocarcinomas per section in AOM-treated WT, *Chrm3*^*-/-*^, *Chrm1*^*-/-*^ and dual KO mice.

Mice treated with vehicle alone did not develop colon neoplasia (not shown). Representative photographs in Figure 2A show robust colon tumor formation in AOM-treated mice. At the 20-week end-point, only colons from *Chrm3*^*-/-*^ mice had reduced tumor burden compared to WT mice (Figure 2A). Tumor measurements revealed 28 and 74% reduction, respectively, in tumor number and size for *Chrm3*^*-/-*^ compared to WT mice (*P* < 0.05 and *P* < 0.005, respectively) (Figure 2B and C), consistent with our previous work [19]. When colon tumors were stratified by volume (Figure 2D), a shift in tumor size in *Chrm3*^*-/-*^ mice towards smaller lesions (< 2 mm^3^) became evident.

We were surprised to observe that genetic ablation of *Chrm1* did not alter the number or volume of colon tumors in *Chrm1*^*-/-*^ mice compared to WT mice (Figure 2A-D). Even more surprising were the outcomes in dual KO mice; tumor number and volume were only slightly reduced compared to those in WT and *Chrm1*^*-/-*^ mice (Figure 2A-D). That is, concomitant genetic ablation of *Chrm1* in *Chrm3*^*-/-*^ mice appeared to mitigate reductions in both colon tumor number and volume that we repeatedly observed in AOM-treated *Chrm3*^*-/-*^ compared to WT mice (Figure 2A-D) [19].

As shown in Figure 2E, in WT, *Chrm1*^*-/-*^ and dual KO mice the majority of colon tumors were adenocarcinomas. In contrast, *Chrm3*^*-/-*^ mice had nearly equivalent numbers of adenomas and adenocarcinomas. Although adenomas were numerically less frequent in dual KO compared to WT mice (Figure 2E), this was not a significant difference (*P* = 0.1). Likewise, there were no significant differences in the numbers of adenocarcinomas per section when comparing *Chrm1*^*-/-*^, dual KO and WT mice. Conversely, the 76% reduction in adenocarcinomas in colons from *Chrm3*^*-/-*^ compared to WT mice was highly significant (*P* < 0.001) (Figure 2E). These findings suggest that the major impact of M3R deficiency in AOM-treated mice is to block progression of colon adenomas to adenocarcinomas.

We evaluated the multiplicity of adenocarcinomas per section; 56% of *Chrm3*^*-/-*^ mouse colons had no adenocarcinomas and only one *Chrm3*^*-/-*^ mouse had more than one colon adenocarcinoma. In contrast, more than 50% of WT, *Chrm1*^*-/-*^ and dual KO mice had multiple (two to seven) adenocarcinomas per section and only two of 20 WT (10%) and one of 12 dual KO (8%) mice had no adenocarcinomas (*P* < 0.01 for reduced multiplicity of tumors in colons from *Chrm3*^*-/-*^ mice vs. colons from the other three genotypes). Colons from all *Chrm1*^*-/-*^ mice contained at least one adenocarcinoma.

We considered the possibility that modifying our protocol to delay AOM treatment of frail dual KO mice until they were 12 weeks old might have impacted outcomes – AOM treatment in the three other genotypes started when mice were six weeks old. To exclude this as a confounder, we started AOM treatment in WT mice at six (N = 25) or 12 (N = 16) weeks of age. Twenty weeks after starting AOM treatment there was no difference in tumor number or volume when comparing mice that started AOM treatment at age six versus 12 weeks (Additional file 1). We concluded that the failure to observe reduced tumor number and size in AOM-treated dual KO mice cannot be attributed to the delay in initiating AOM treatment.

### Effect of M1R, M3R and dual knockout on tumor cell proliferation and apoptosis

**Figure 3:**
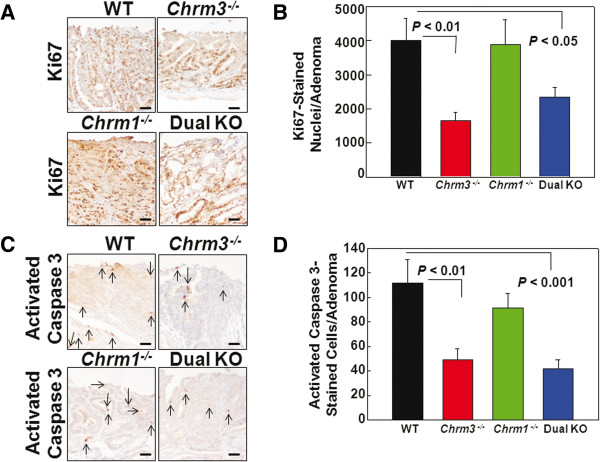
**Markers of cell proliferation and apoptosis in colon adenomas from AOM-treated mice. A**. Representative staining for Ki67 in adenomas from WT, *Chrm3*^*-/-*^, *Chrm1*^*-/-*^ and dual KO mice. **B**. Ki67 staining was significantly reduced in adenomas from *Chrm3*^*-/-*^ and dual KO compared to WT mice. **C**. Representative staining for activated caspase-3 in adenomas from WT, *Chrm3*^*-/-*^, *Chrm1*^*-/-*^ and dual KO mice. Stained cells are indicated by arrows. **D**. Activated caspase-3 staining was significantly reduced in adenomas from *Chrm3*^*-/-*^ and dual KO compared to WT mice. For *B* and *D*, N = 22 WT, 19 *Chrm3*^*-/-*^, 23 *Chrm1*^*-/-*^ and 12 dual KO mice. Size bars = 200 micrometers.

To determine whether changes in tumor number and size resulted from differences in cell proliferation and apoptosis, we examined Ki67 and activated caspase-3 staining, respectively. Figure 3A shows representative micrographs of Ki67 staining in adenomas from AOM-treated WT, *Chrm3*^*-/-*^, *Chrm1*^*-/-*^ and dual KO mice. Compared to adenomas from AOM-treated WT mice, Ki67 staining was significantly reduced in those from *Chrm3*^*-/-*^ and dual KO but not *Chrm1*^*-/-*^ mice (Figure 3). Figure 3B shows 58% reduction in Ki67-positive cells in adenomas from *Chrm3*^*-/-*^ compared to those from WT mice (*P* < 0.01). Ki67 staining was reduced 42% in dual KO mice (*P* < 0.05 compared to WT mice). Thus, although Ki67 staining was significantly reduced in dual KO compared to WT mice, this reduction was less than that observed in *Chrm3*^*-/-*^ mice (Figure 3B), suggesting that in AOM-treated mice concomitant ablation of *Chrm1* mitigates anti-proliferative effects of *Chrm3* gene ablation.

Figure 3C shows representative micrographs of activated caspase-3 staining in adenomas from AOM-treated WT, *Chrm3*^*-/-*^, *Chrm1*^*-/-*^ and dual KO mice. The number of apoptotic cells in adenomas from AOM-treated mice was almost two orders of magnitude lower than the number of proliferating cells (compare scales for vertical axes in Figure 3B and D). Apoptotic cells were reduced in adenomas from *Chrm3*^*-/-*^ and dual KO compared to WT mice (*P* < 0.01 and < 0.001, respectively). In contrast, apoptotic cells were not significantly different in adenomas from *Chrm1*^*-/-*^ and WT mice (Figure 3D).

### Genes differentially expressed in colon tumors from WT and *Chrm3*^*-/-*^ mice

Tissues obtained in these experiments provided an opportunity to identify novel genes and signaling pathways potentially underlying tumor-promoting actions of M3R activation in the colon [6,11-14]. We designed an experimental strategy using gene microarray to compare gene expression profiles in tumors from *Chrm3*^*-/-*^ and WT mice, and thereby identify novel candidate M3R-regulated genes worthy of further exploration. To exclude changes in gene expression that resulted from neoplastic transformation but were not a specific consequence of changes in M3R expression, we used qPCR to validate key microarray findings and compare expression of candidate genes in tumors from *Chrm3*^*-/-*^, *Chrm1*^*-/-*^, dual KO and WT mice. We reasoned that genes whose expression levels were different in tumors from *Chrm3*^*-/-*^ compared to WT mice, but not in tumors from *Chrm1*^*-/-*^ compared to WT mice, would more likely be relevant to M3R-induced colon tumor promotion. Although this approach has potential limitations we thought it would provide useful information regarding potential molecular pathways underlying the different tumor phenotypes.

We used an Illumina gene microarray to compare expression profiles for 19,100 genes in tumors from *Chrm3*^*-/-*^ and WT mice. Using 474 probes, we identified 430 genes either up- or down-regulated by enrichment scores of at least 1.3 (equivalent to a non-log scale value of 0.05) in tumors from AOM-treated *Chrm3*^*-/-*^ mice compared to those from WT mice. Ingenuity Pathway Analysis of this data set did not reveal involvement of a known pathway to explain tumor attenuation in AOM-treated *Chrm3*^*-/-*^ compared to WT mice (data not shown).

**Figure 4:**
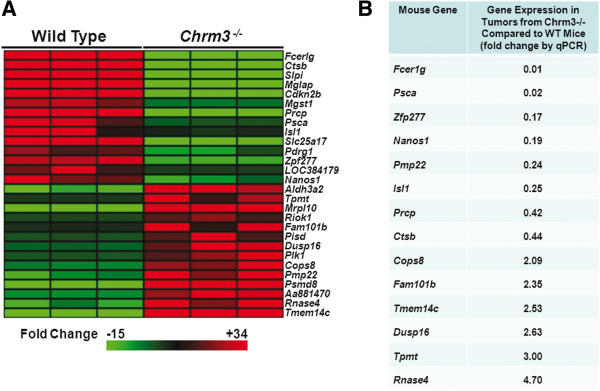
**Analyses of gene expression in tumors from WT and *Chrm3***^***-/-***^**mice. A**: Heat map displaying expression pattern of the 28 genes with the largest changes in expression when comparing tumors from *Chrm3*^*-/-*^ relative to tumors from WT mice. Rows represent genes and columns represent samples. Color intensity denotes the standardized ratio between each value and the average expression of each gene across all samples. Red pixels indicate increased abundance and green pixels decreased abundance of each transcript in the indicated sample. N = 3 mice/ genotype. **B**: Comparison of changes in murine gene expression levels in tumors from *Chrm3*^*-/-*^ mice relative to tumors from WT mice quantified by real time RT-PCR (qPCR). WT, wild-type.

Surmising that M3R-regulated genes important for colon tumor promotion would undergo relatively large changes in expression, for further analysis we arbitrarily set a cut-off at the top 14 down-regulated and top 14 up-regulated genes identified by microarray in tumors from *Chrm3*^*-/-*^ compared to WT mice, a total of 28 genes (Figure 4A). The heat map deriving from this ‘validation set’ demonstrates consistent changes in gene expression in tumors from three mice from each genotype. There were large changes in expression of some genes. *Fcer1g* gene expression was nearly abolished in tumors from *Chrm3*^*-/-*^ compared to those from WT mice whereas *Tmem14C* gene expression was strongly up-regulated (Figure 4A), findings confirmed by qPCR using the same mRNA employed for the microarray analysis (not shown).

We used qPCR to examine expression of a subset of genes strongly associated with colon neoplasia [25]. *Cyclin D1* expression was unchanged in tumors from *Chrm3*^*-/-*^ mice compared to those from WT mice suggesting that changes in some genes key to intestinal neoplasia occurred prior to the 20-week end-point (Additional file 2). Nonetheless, at the 20-week time-point, expression of both *c-myc* and *β-catenin* in tumors from *Chrm3*^*-/-*^ compared to those from WT mice was reduced 25%, and *Myd88* expression was reduced 32% (Additional file 2). The greatest changes were in *Egfr* and *Cox2* expression, reduced 54 and 70%, respectively, in tumors from *Chrm3*^*-/-*^ mice (Additional file 2). Altered *Egfr* expression was of particular interest since we showed previously that cross-talk between M3R and EGFR is critical for human colon cancer cell proliferation [6] and others showed that Egfr signaling in mice is required for AOM-induced colon neoplasia [26,27]. *COX2* is also over-expressed in human colon neoplasia [28,29] and, in mouse models of intestinal neoplasia, *Cox2 (Ptgs2)*-deficiency attenuates tumor formation [30]. We also confirmed the absence of compensatory increases in *Chrm3* expression in *Chrm1*^*-/-*^ mice that could account for the *Chrm1*^*-/-*^ colon tumor phenotype.

To validate changes in expression of the 28 genes shown in Figure 4A, we extracted additional mRNA from colon tumors from WT and *Chrm3*^*-/-*^ mice and compared gene expression using qPCR. To more effectively separate signal from noise, for this analysis we selected an arbitrary conservative threshold of two-fold or greater changes in mRNA expression. Applying these more stringent criteria, we detected meaningful changes in the expression of mRNA for 14 genes derived from the original 28-gene set (Figure 4A); eight genes were down-regulated and six were up-regulated in tumors from *Chrm3*^*-/-*^ compared to WT mice (Figure 4B). To our knowledge, none of the 14 genes in this ‘core *Chrm3*-dependency gene set’ were previously associated with muscarinic receptor signaling.

### Comparison of gene expression in colon tumors from *Chrm3*^*-/-*^, *Chrm1*^*-/-*^, dual KO and WT mice

**Figure 5:**
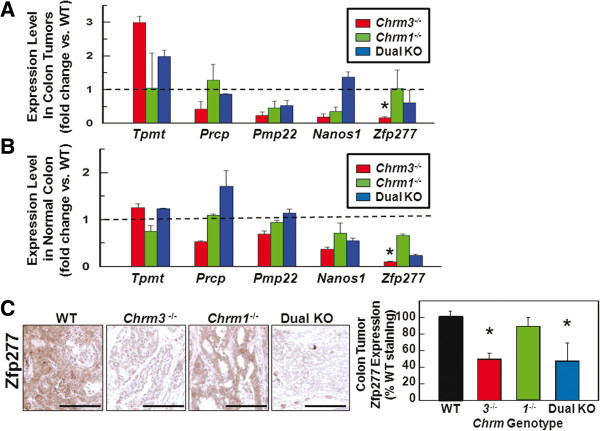
**Expression levels of murine genes quantified by qPCR in tumor and normal colon tissue, and Zfp277 protein expression in tumor sections from WT, *Chrm3***^***-/-***^**, *Chrm1***^***-/-***^**and dual KO mice.A**: Colon tumors. N = 3 mice/genotype. **B**: Normal colon. N = 3 mice/genotype. Shown is the fold difference in gene expression normalized to glyceraldehyde-3-phosphate dehydrogenase (*Gapdh*) levels in tissue from *Chrm3*^*-/-*^, *Chrm1*^*-/-*^ and dual KO mice relative to that in tissue from WT mice. Error bars, S.E. (* indicates *P* < 0.05). **C**: Representative images show immunohistochemical staining with anti-Zfp277 antibody on tumor sections from WT, *Chrm3*^*-/-*^, *Chrm1*^*-/-*^ and dual KO mice. Graph shows quantification of Zfp277 staining in tumor sections. Data shown represent mean ± S.E. from four mice per genotype (* indicates *P* < 0.05). Size bars = 100 micrometers.

To identify novel genes relevant to *Chrm* subtype-dependent differences in colon neoplasia, we used qPCR to measure expression of the core *Chrm3*-dependency gene set shown in Figure 4B using mRNA extracted from tumors from *Chrm3*^*-/-*^, *Chrm1*^*-/-*^ and dual KO mice, and then compared expression of these 14 genes to that in tumors from WT mice. Specifically, we sought genes whose expression levels in tumors from *Chrm3*^*-/-*^ and *Chrm1*^*-/-*^ mice compared to those from WT mice were consistent with the different colon neoplasia phenotypes (Figure 2B and C); that is, we thought it important to pursue genes with meaningful changes in expression in tumors from *Chrm3*^*-/-*^ compared to WT mice, but not in tumors from *Chrm1*^*-/-*^ compared to WT mice. Using this approach we identified five candidate mouse genes meeting these criteria - *Tpmt, Prcp, Pmp22, Nanos1* and *Zfp277.* One gene (*Tpmt*) was up-regulated in tumors from *Chrm3*^*-/-*^ mice and four (*Prcp*, *Pmp22*, *Nanos1* and *Zfp277*) were down-regulated (Figure 5A). However, the only change in gene expression that achieved statistical significance was reduced *Zfp277* levels in tumors from *Chrm3*^*-/-*^ compared to those from WT and *Chrm1*^*-/-*^ mice (*P* < 0.05, Figure 5A).

Next, to determine whether similar changes in gene expression occurred in non-neoplastic colon, we used qPCR to compare expression levels of these five candidate genes in normal colon from WT, *Chrm3*^*-/-*^, *Chrm1*^*-/-*^ and dual KO mice that had not been treated with either AOM or vehicle (Figure 5B). In these experiments, we used expression of an endogenous housekeeping gene, *Gapdh*, as an internal control; *Gapdh* expression did not change significantly in normal colon and tumors across mouse genotypes (not shown). Comparison of the data in Figure 5A and B revealed similar patterns of gene expression in tumors and normal colon tissue for *Prcp, Pmp22, Nanos1* and *Zfp277*, but not for *Tpmt*. Hence, we considered it likely that changes in expression of mRNA for *Tpmt* in tumors resulted from neoplastic transformation irrelevant to regulation by muscarinic receptor signaling; *Tpmt* was excluded from further consideration. Again, the only change in gene expression achieving statistical significance was reduced *Zfp277* levels in normal colon from *Chrm3*^*-/-*^ compared to expression in normal colon from WT and *Chrm1*^*-/-*^ mice (*P* < 0.05, Figure 5B). Based on these collective findings, we selected *Zfp277* for further analysis and excluded *Tpmt, Prcp*, *Pmp22* and *Nanos1* from further consideration as genes relevant to M3R-regulated colon neoplasia.

### Zinc finger protein 277 expression in tumors from WT, M1R- and M3R-deficient mice

To establish the functional relevance of changes in gene expression, we used immunohistochemistry with specific antibodies to interrogate colon tumor sections for changes in Zfp277 protein expression corresponding to changes in *Zfp277* mRNA levels. In colon tumors from WT mice we observed robust Zfp277 staining (Figure 5C). Notably, Zfp277 protein expression was reduced approximately 50% in tumors from *Chrm3*^*-/-*^ and dual KO mice compared to tumors from WT mice (*P* < 0.05) (Figure 5C). As anticipated from the mRNA data, Zfp277 protein expression was the same in tumors from WT and *Chrm1*^*-/-*^ mice (Figure 5C). Taken together, these novel findings identify parallel changes in both gene and protein expression for Zfp277.

### Zinc finger protein 277 is over-expressed in human colon cancer

As an initial exploration of the potential clinical significance of these findings, we searched human expression data sets in the National Center for Biotechnology Information (NCBI) Gene Expression Omnibus (GEO) Profiles database. Specifically, in datasets of human colon cancer compared to adjacent normal colon tissue from the same person we sought information regarding the expression of *ZNF277*, the human analogue of *Zfp277*. This database search yielded only one relevant study of surgical specimens comparing gene expression profiles in colon cancer compared to normal colon [31]. Our *in silico* analysis of this existing dataset revealed that *ZNF277* expression was significantly up-regulated in 33 of 34 colon cancers compared to adjacent normal colon (*P* = 2 × 10^-8^) [31].

**Figure 6:**
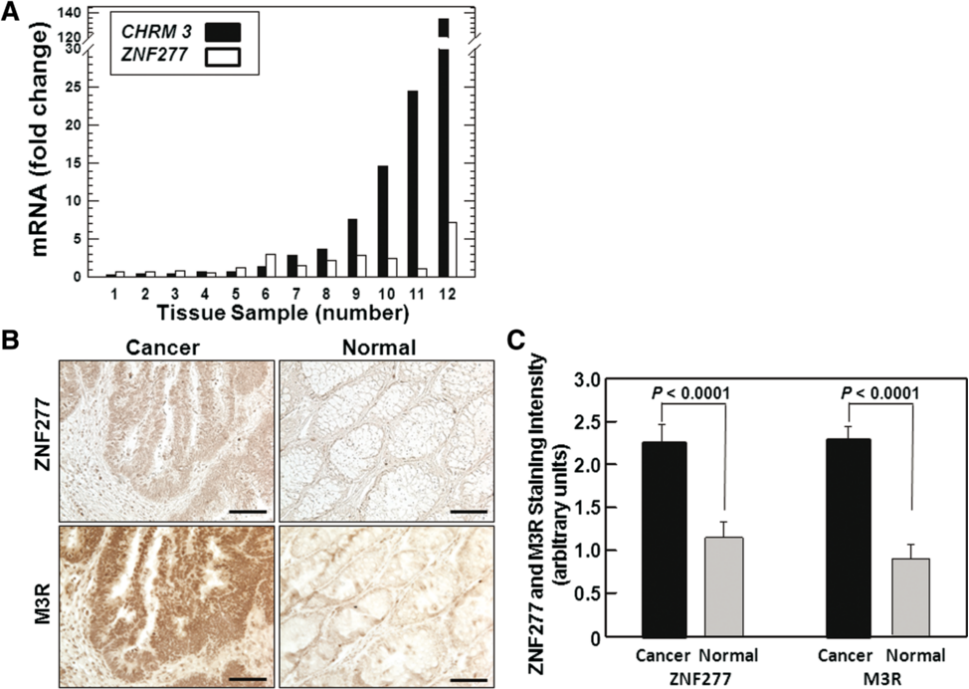
**M3R and ZNF277 gene and protein expression in human colon cancer compared to adjacent normal colon. A**: *CHRM3* and *ZNF277* mRNA levels in 12 sets of colon cancer and adjacent normal colon obtained from the same patients. mRNA levels were first normalized to expression of *β2-microglobulin* and then ranked by increasing levels of *CHRM3* expression. **B**: Representative images show immunohistochemical staining with anti-ZNF277 and anti-M3R antibodies on human colon cancer and adjacent normal colon epithelial tissue. Size bars = 100 micrometers. **C**: Graph shows quantification of ZNF277 and M3R staining in cancer compared to normal colon. Data shown represent mean ± S.E. of 23 sets of colon cancer and adjacent normal colon tissues.

We sought to confirm this observation in our lab by interrogating a set of 12 archived colon cancer tissues with adjacent normal colon that had been flash-frozen immediately after surgery at our own institution and stored at -80°C. In Figure 6A, changes in mRNA expression in colon cancer compared to normal colon are depicted in order of increasing *CHRM3* expression (black bars). Although increases in *ZNF277* mRNA were not as robust as those for *CHRM3* mRNA, the expression patterns were very similar (Figure 6A). In these 12 human tissue samples, over-expression of *ZNF277* in colon cancer was contingent upon over-expression of *CHRM3* (Spearman rank correlation coefficient = 0.73).

Next, we validated the importance of these changes in gene expression by measuring and comparing M3R and ZNF277 protein expression in an archived set of 23 formalin-fixed paraffin-embedded colon cancer tissues with adjacent normal colon from the same patients. We analyzed these samples immunohistochemically using specific antibodies for M3R and ZNF277 (Figure 6B). As shown in Figure 6C, zinc finger protein 277 and M3R were both significantly over-expressed in cancer compared to adjacent normal tissue. Moreover, as we observed for gene expression, statistical analysis of these human tissue data indicated that over-expression of ZNF277 and M3R protein were also contingent variables; i.e., ZNF277 over-expression was statistically associated with M3R over-expression (*P* = 0.04). Collectively, these data support the novel conclusion that in both murine and human colon cancer, zinc finger protein 277 plays a currently undefined role in M3R-mediated promotion of neoplasia.

## Discussion

Experimental mouse models that mimic clinical and molecular aspects of human colon cancer progression are useful tools to identify novel mechanisms underlying colon neoplasia [24]. Here, to explore the individual roles and interplay between two muscarinic receptor subtypes, we analyzed systematically the effects of *Chrm3* and *Chrm1* gene ablation, alone and in combination, on AOM-induced murine colon neoplasia, a model closely resembling non-hereditary human colon cancer [24]. To gain additional genetic and potentially mechanistic insights, we used a hypothesis-unbiased approach to elucidate concomitant changes in gene expression levels in tumors from WT and M3R-deficient mice.

As anticipated from our previous work [19,20], tumor number and size as well as tumor cell proliferation measured by Ki67 staining were greatly attenuated in AOM-treated *Chrm3*^*-/-*^ compared to WT mice. Based on our previous findings regarding the interplay of M1R and M3R in gastric function [21], we hypothesized that compared to the effects of *Chrm3* gene ablation alone, concurrent ablation of *Chrm3* and *Chrm1* might further attenuate, or even abolish, AOM-induced tumor formation. Instead, we were surprised to observe that *Chrm1* gene ablation alone did not alter tumor formation. We were further surprised to observe that combined *Chrm3* and *Chrm1* knockout mitigated reductions in tumor number and size observed with *Chrm3* knockout alone.

We can draw several novel conclusions from these observations. Whereas work from our [19] and other groups [2-4,7,8] provide strong evidence that M3R acts as a tumor promoter, the present observations newly suggest that in the colon M1R acts as a tumor suppressor. This putative role for M1R is unmasked by combined M1R and M3R deficiency in dual KO mice where tumor formation is similar to that observed in WT mice. Thus, M1R deficiency appears to negate the beneficial effects of knocking out only M3R. This finding may also explain why treating *Apc*^*min/+*^ mice with a non-selective muscarinic receptor inhibitor, scopolamine butylbromide, attenuates intestinal tumor formation less efficaciously than M3R gene ablation [20]. Scopolamine butylbromide treatment blocks both M3R and M1R activation, thereby mimicking combined M3R and M1R deficiency in dual KO mice. These considerations intimate therapeutic promise for a pharmacological approach to block M3R activation while at the same time augmenting M1R activation.

The unanticipated findings regarding the impact of *Chrm3* and *Chrm1* knockout on AOM-induced colon neoplasia provided an opportunity to explore M3R-regulated changes in tumor gene expression. We identified a set of genes expressed differentially in tumors from WT and M3R-deficient mice. Then, we used tumors from M1R-deficient mice as controls to exclude non-specific changes in gene expression due to neoplastic transformation but not a specific consequence of altered M3R expression. We employed a gene microarray comprising 19,100 target genes to identify 430 genes with expression levels significantly altered in tumors from M3R-deficient compared to WT mice (strategy schematized in Additional file 3); a dataset further refined by increasing statistical stringency and using qPCR to validate results. These combined approaches identified 14 promising genes with meaningful changes in expression in tumors from M3R-deficient compared to WT mice (Figure 4B).

We concede this gene discovery approach has limitations. It may have identified only a fraction of the genome; many genes are turned off or encode proteins required for survival in specific amounts that do not change. Also, protein expression may be regulated by mechanisms that do not involve altered mRNA levels. Financial constraints limited expression profiling experiments to a relatively small number of observations under identical conditions and for the same reason limited further investigation of candidate genes to the relatively small subset of 14 genes shown in Figure 4B, thereby reducing statistical power. We may have missed important but subtle changes in gene expression. Even so, we believe that confirmatory results from our qPCR experiments provided reliable measures of changes in the expression levels of this 14-gene subset in both tumors and normal colon from WT, *Chrm3*^*-/-*^, *Chrm1*^*-/-*^ and dual KO mice.

Changes in expression of only one gene, *Zfp277*, achieved statistical significance with matching changes in expression of the corresponding protein. Based on the stringent overall approach (Additional file 3), we are confident that our work identifies a novel role for *Zfp277* as an M3R-regulated gene pertinent to the progression of intestinal neoplasia. Confidence in this conclusion was bolstered by detecting over-expression of both mRNA and protein for ZNF277, the human analogue of *Zfp277*, in human colon cancer samples and, importantly, that this over-expression mirrored that of *CHRM3* and M3R.

Literature and gene bank searches revealed little regarding the function of *ZNF277* (*NIRF4*), which is expressed in multiple tissues, including the proximal colon [32]. The protein product, zinc finger protein 277, is reported to play a role in cellular senescence and protection against genomic instability and cancer [33]. *ZNF277* over-expression is reported in other cancers – chronic lymphocytic leukemia, well-differentiated renal cell carcinoma, and germ cell and endocrine tumors [32]. Hence, a novel, hitherto unrecognized role for *ZNF277* in colon cancer biology is certainly plausible. We found no previous reports of an association between either *Zfp277* or *ZNF277* expression and muscarinic receptor expression or activation. In future work, we plan to use *in vitro* and *in vivo* models to explore the molecular mechanisms underlying the association between M3R and ZNF277 expression.

## Conclusions

Our results identify a novel candidate mouse gene, *Zfp277,* whose expression pattern is compatible with a role in mediating divergent effects of *Chrm3* and *Chrm1* gene ablation on murine intestinal neoplasia. Although finding an association between ZNF277 and M3R over-expression in human colon cancer is reassuring, we do not currently understand the molecular mechanism underlying this interaction. Future work will use both *in vitro* and *in vivo* approaches to address these questions and elucidate the functional role of *ZNF277* and its interaction with M3R in colon cancer. Although it is currently unclear why M3R and M1R, which both signal by stimulating phospholipid turnover and changes in cell calcium [10], have such divergent effects on colon neoplasia, their contrary roles suggest that jointly targeting these receptors may have therapeutic potential. Based on these considerations, we are optimistic that improved understanding of the role of muscarinic receptors in neoplasia will continue to yield novel therapeutic targets for colon cancer.

## Methods

### Animals

*Chrm3*^*-/-*^ and *Chrm1*^*-/-*^ mice were generated from the same mixed genetic background (129S6/SvEvTac X CF1: 50%/50%) as described previously [23,34,35]. Dual KO mice on the same genetic background were generated by mating homozygous *Chrm1*^*-/-*^ and *Chrm3*^*-/-*^ mutant mice [36]. For all experiments, only male mice were used and aged-matched WT mice of the same genetic background served as controls. Mice were housed under identical conditions in a pathogen-free room, had free access to commercial rodent chow and water, and were acclimatized in the vivarium for at least one week before experiments. These studies were approved by the University of Maryland School of Medicine Institutional Animal Care and Use, and the Baltimore VA Research and Development Committees.

### Human tissues

To examine M3R (*CHRM3*) and ZNF277 gene and protein expression, we used archived pre-existing de-identified surgical specimens of colon cancer and adjacent normal colon epithelium (approved by the University of Maryland School of Medicine Institutional Review Board and the Baltimore VA Research and Development Committee).

### Study design

For the initial 6 weeks of treatment, 94 mice (25 WT, 20 *Chrm3*^*-/-*^, 23 *Chrm1*^*-/-*^ and 26 dual KO mice) received weekly intraperitoneal injections of azoxymethane (AOM; Midwest Research Institute; 10 mg/kg body weight) and 21 mice (5 WT, 5 *Chrm3*^*-/-*^, 5 *Chrm1*^*-/-*^ and 6 dual KO mice) received an equal volume of vehicle (phosphate-buffered saline) (Figure 1A). As in our previous study [19], AOM and vehicle treatment in WT, *Chrm3*^*-/-*^ and *Chrm1*^*-/-*^ mice was started when animals were 6-weeks old. In dual KO mice, AOM and PBS treatments were initiated at 12 weeks of age. All animals were euthanized 20 weeks after initiating AOM injections. Colon length was measured, and segments were opened longitudinally and placed flat on microscope slides. Tumors were identified by visual inspection and photographed (Nikon SMZ1500 dissecting microscope). Tumor size was measured using calipers and tumor volume calculated using: volume = ½ (length × width^2^) [37].

### Histological and immunohistochemical staining analysis

Tissues were fixed in 4% paraformaldehyde and paraffin-embedded. Five-micrometer sections were stained with hematoxylin and eosin. Adenomas and adenocarcinomas were defined according to consensus recommendations by the Mouse Models of Human Cancers Consortium [38]. As markers of cell proliferation and apoptosis, we used immunohistochemical staining for Ki67 and activated caspase-3, respectively (antibodies from Cell Signaling Technology). Only complete crypts were evaluated and investigators were masked to genotype and treatment group.

To identify corresponding changes in protein expression for relevant genes identified by microarray and qPCR, formalin-fixed paraffin-embedded tumor sections were immunostained with a specific antibody against both mouse Zfp277 and human ZNF277 from Santa Cruz Biotech (Santa Cruz, CA), and a specific antibody from Alomone Labs (Jerusalem, Israel) against both mouse and human M3R. Tumor sections were examined with a Nikon 80*i* photomicroscope at 200× magnification. Sections were first reviewed and scored by a senior pathologist (CD) masked to tissue origin and immunostaining was then quantified using Image-Pro Plus software (version 5.1; Media Cybernetics, Silver Spring, MD). To minimize variation, all tumor sections were examined and photographed using the same microscope settings.

### Microarray performance and analyses

After resection, murine tissue was immediately stored in *RNAlater* (Ambion) at -80°C. Total RNA was extracted using the RNeasy kit from Qiagen. RNA was digested using the RNase-Free DNase set. The quality of total RNA was tested and confirmed using a Bioanalyzer 2100 (Expression Analysis, Inc., Durham, NC). The microarray assay was performed by Expression Analysis, Inc. using the MouseWG-6 v2.2 Expression BeadChip (Illumina, San Diego, CA). This chip covers the whole mouse genome, > 19,100 unique, curate genes targeting a total of 45,281 transcripts. Results were analyzed using the cubic spline normalization method without background subtraction [39,40]. In comparing changes in mRNA expression in *Chrm3*^*-/-*^ vs. WT mouse colon tumors, statistical significance cutoff levels were set for individual transcripts at *P* ≤ 0.05 (false discovery rate) and enrichment scores ≥ ± 1.3; changes in gene mRNA that met these thresholds were deemed to be differentially expressed. Microarray data represent results of tissue from three different mice per genotype. Results were submitted to the National Center for Biotechnology Information (NCBI) Gene Expression Omnibus (GEO) database (GSE43444).

### Quantitative RT-PCR (qPCR)

First-strand cDNAs were synthesized from 5 μg RNA (Superscript III First Strand Synthesis System for RT-PCR, Invitrogen). qPCR was then performed using 50 ng cDNA, the SYBR Green PCR Master Mix (Applied Biosystems), and forward and reverse primers (final concentration 0.5 μM in sample volumes of 20 μl). Primers (Additional file 4) were designed to span introns using the National Center for Biotechnology Information nucleotide database SIM-4 gene alignment program and on-line software (http://www.genscript.com/index.html). qPCR was performed using the 7900HT Fast System (ABI) with Power SYBR Green Master Mix (ABI). PCR conditions included 5 min at 95°C followed by 37 cycles of 95°C for 15 seconds, 60°C for 20 seconds, and 72°C for 40 seconds and a final cycle at 95°C for 15 seconds, 60°C for 15 seconds, and 95°C for 15 seconds. PCR data were analyzed using ABI instrument software SDS 2.1. Expression of candidate genes in each group of mice was normalized to *glyceraldehyde 3-phosphate dehydrogenase* (*Gapdh*). For human samples, expression of *CHRM3* and *ZNF277* was normalized to *β*_*2*_*-microglobulin* (*B2M*), a preferable housekeeping gene for analysis of colon cancer [41]. Quantitative qPCR data were evaluated using the comparative *C*_T_ (2^–ΔΔ*C*T^) method [42].

### Statistical analysis

Student’s unpaired *t*-test was used to determine statistical significance. The strength of linear association between two variables was quantified using the Spearman’s rank correlation coefficient and that of non-linear associations by Pearson’s chi-squared test. *P* values ≤0.05 were considered significant.

## Competing interests

The authors declare that they have no competing interests.

## Authors’ contributions

KC, GX, SK and JPR conceived the study design and designed the experiments. KC, SK, JT, NS and JPR carried out the study. JPR prepared the manuscript. KC, SK and JPR supervised the statistical analysis. KC, GX, SK, CD and JPR revised the manuscript. KC, JH and CD performed the histopathological assessments of animal and human tissue. KC, GX, SK and JPR supervised the interpretations of results and revised the manuscript. All authors read and approved the final manuscript.

## Supplementary Material

Additional file 1 **Delaying initiation of AOM treatment until mice are 12-weeks-old rather than 6-weeks-old does not alter colon tumor number.** Comparison of tumor number (left panel) and tumor volume (right panel) 20 weeks after starting AOM treatment in 6- and 12-week-old WT mice (mean ± S.E.). N represents number of mice per treatment group. Click here for file

Additional file 2 **Comparison of changes in expression of key genes associated with colon neoplasia in tumors from Chrm3**^**-/-**^** mice relative to tumors from WT mice quantified by real time RT-PCR (qPCR).** Click here for file

Additional file 3 **Experimental strategy used to identify changes in gene and protein expression relevant to divergent effects of muscarinic receptor gene ablation on colon neoplasia phenotypes.** The experimental approaches, comparators and yields (outcomes) for each stage of investigation are outlined. An enrichment score of 1.3 is equivalent to a non-log scale value of 0.05. qPCR, quantitative RT-PCR; WT, wild-type; IHC, immunohistochemical analysis. Click here for file

Additional file 4 RT-PCR (qPCR) primers used in this study. Click here for file
