# Supplementary figures and images for: Divergent effects of muscarinic receptor subtype gene ablation on murine colon tumorigenesis reveals association of M3R and zinc finger protein 277 expression in colon neoplasia
Source: Mol Cancer. 2014 Apr 3;13:77. doi: 10.1186/1476-4598-13-77 (PMC4021221; doi:10.1186/1476-4598-13-77)

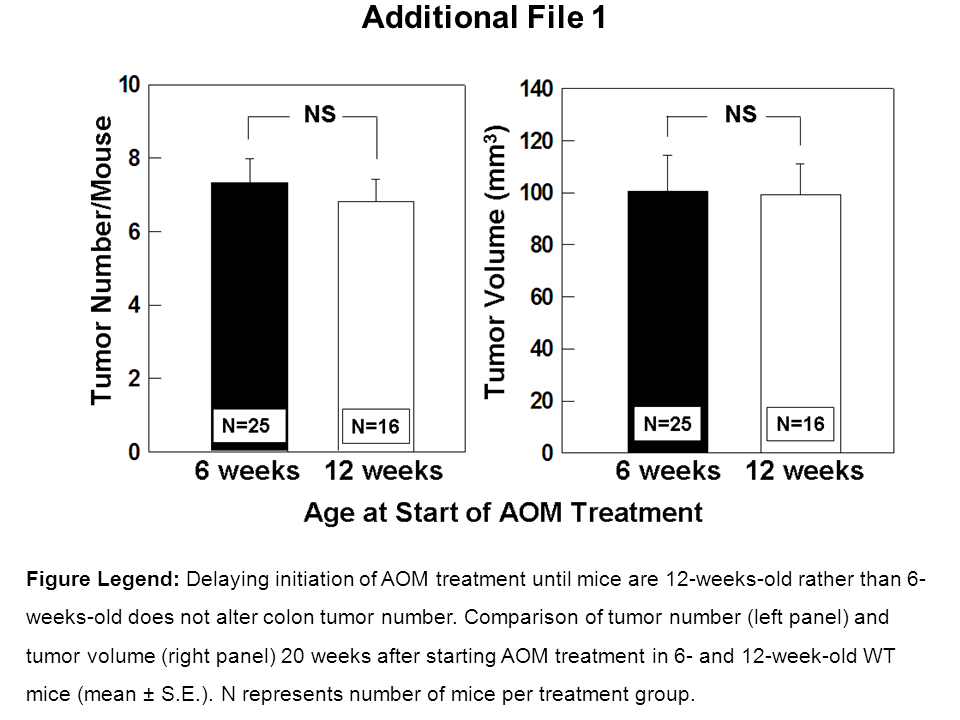

Supplement: Additional file 1 — Delaying initiation of AOM treatment until mice are 12-weeks-old rather than 6-weeks-old does not alter colon tumor number. Comparison of tumor number (left panel) and tumor volume (right panel) 20 weeks after starting AOM treatment in 6- and 12-week-old WT mice (mean ± S.E.). N represents number of mice per treatment group. [file 1476-4598-13-77-S1.tiff]

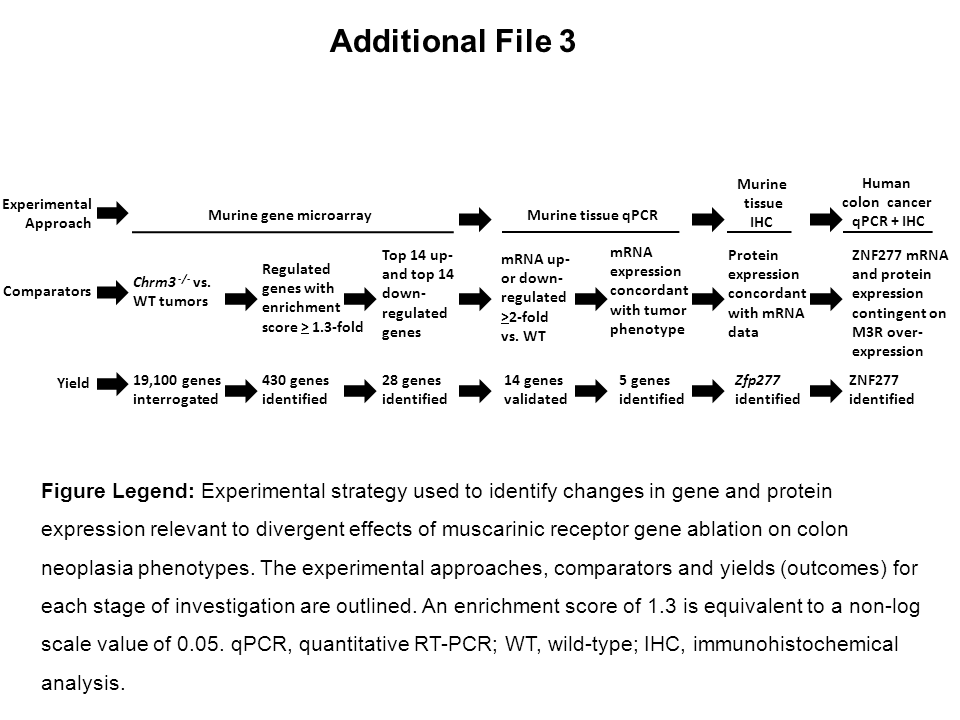

Supplement: Additional file 3 — Experimental strategy used to identify changes in gene and protein expression relevant to divergent effects of muscarinic receptor gene ablation on colon neoplasia phenotypes. The experimental approaches, comparators and yields (outcomes) for each stage of investigation are outlined. An enrichment score of 1.3 is equivalent to a non-log scale value of 0.05. qPCR, quantitative RT-PCR; WT, wild-type; IHC, immunohistochemical analysis. [file 1476-4598-13-77-S3.tiff]
