# Supplementary material for: Divergent effects of muscarinic receptor subtype gene ablation on murine colon tumorigenesis reveals association of M3R and zinc finger protein 277 expression in colon neoplasia
Source: Mol Cancer. 2014 Apr 3;13:77. doi: 10.1186/1476-4598-13-77 (PMC4021221; doi:10.1186/1476-4598-13-77)
Supplement: Additional file 2 — Comparison of changes in expression of key genes associated with colon neoplasia in tumors from Chrm3 -/- mice relative to tumors from WT mice quantified by real time RT-PCR (qPCR). [file 1476-4598-13-77-S2.doc]

**Additional File 2.** Comparison of changes in expression of key genes associated with colon neoplasia in tumors from Chrm3^-/-^ mice relative to tumors from WT mice quantified by real time RT-PCR (qPCR)

| **Mouse Gene** | **Symbol** | **Gene Expression in Tumors from *Chrm3^-/-^* Compared to WT Mice (fold-change by qPCR)** |
| --- | --- | --- |
| *CyclinD1* | Ccnd1 | 1.03 |
| *c-myc* | Myc | 0.75 |
| *β-catenin* | Ctnnb1 | 0.75 |
| *Myd88* | Myd88 | 0.68 |
| *Egfr* | Egfr | 0.46 |
| *Cox-2* | Ptgs2 | 0.30 |

WT, wild-type
