# Supplementary material for: Divergent effects of muscarinic receptor subtype gene ablation on murine colon tumorigenesis reveals association of M3R and zinc finger protein 277 expression in colon neoplasia
Source: Mol Cancer. 2014 Apr 3;13:77. doi: 10.1186/1476-4598-13-77 (PMC4021221; doi:10.1186/1476-4598-13-77)
Supplement: Additional file 4 — RT-PCR (qPCR) primers used in this study. [file 1476-4598-13-77-S4.doc]

**Additional File 4.** RT-PCR (qPCR) primers used in this study.

| **Mouse Genes** | **Primer Sequence (5’-3’)** | |
| --- | --- | --- |
| *Aa881470* | F | TTGGAGAAACTGCTGGACTG |
|  | R | CTCCATTAAGCAGGGATGGT |
| *Aldh3a2* | F | TTGCAGCTGTGATTGTCAAG |
|  | R | GTGAATTTCCTGGTTTGGGT |
| *Ccnd1* | F | CAACGCACTTTCTTTCCAGA |
|  | R | GACTCCAGAAGGGCTTCAAT |
| *Cdkn2b* | F | CTCCAGCTCGACAAGGAAAT |
|  | R | CAATTCATCACTGGGCTTTG |
| *Cops8* | F | GAGACTGTGCAGCCAATCAT |
|  | R | TTTCACAGCCTCTTCCACAG |
| *Ctnnb1* | F | CCTGAGGGTACCTGAAGCTC |
|  | R | GCTTGAGTAGCCATTGTCCA |
| *Ctsb* | F | ATGACAAGCCTTCCTTCCAC |
|  | R | GACAGTGCCACACAGCTTCT |
| *Dusp16* | F | GTGCCTGTGAATGACAGCTT |
|  | R | TGGATAAGCACACAGCCATT |
| *Egfr* | F | CCACGCCAACTGTACCTATG |
|  | R | CACAATCCCAGTGGCAATAG |
| *Fam101b* | F | TATAGTCCCGAACCCAGCAT |
|  | R | GACAGAGTCTCGGTGAGCAG |
| *Fcer1g* | F | ACTCAAGATCCAGGTCCGAA |
|  | R | AAGCACGTCTGTTCTGAAGC |
| *Gapdh* | F | ACAACTTTGGCATTGTGGAA |
|  | R | GATGCAGGGATGATGTTCTG |
| *Isl1* | F | GCTTTGTTAGGGATGGGAAA |
|  | R | CGAAGTCGTTCTTGCTGAAG |
| *Loc384179* | F | GGGTGCAACTTATGGCAAG |
|  | R | CTCAAAGTCCCACAATGGC |
| *Mglap* | F | TCTCACGAAAGCATGGAGTC |
|  | R | TAGCTCGCCACCTCTGCT |
| *Mgst1* | F | AGTTTGTTCGCACTGACGAG |
|  | R | GTGCATGAGGGCTGTAGAGA |
| *Mrpl10* | F | GAAATGGTGCGGGTCTTAAA |
|  | R | GCTGAGGATGGTGTCATCAA |
| *Myc* | F | CAGCAGCGACTCTGAAGAAG |
|  | R | GACTCCGACCTCTTGGGA |
| *Myd88* | F | TAGAGCTGCTGGCCTTGTTA |
|  | R | GCCACCTGTAAAGGCTTCTC |
| *Nanos1* | F | TGCCTAAGAAAGCACTGTGG |
|  | R | GACAAATGCAGGCTAAAGCA |
| *Pdrg1* | F | GATGTGATGGTTTGCTTTGG |
|  | R | TCCGCAGCCTTTCTATTTCT |

| *Pisd* | F | AAACAATGCCACTGGTTTCC |
| --- | --- | --- |
|  | R | AGCCTGGCTACAGCTTCCTA |
| *Plk1* | F | GAGACCTACCTCCGGATCAA |
|  | R | CTCGTCATTGAGCAACTCGT |
| *Pmp22* | F | GCAGAACTGTACCACATCCG |
|  | R | CTGGCAGAAGAACAGGAACA |
| *Prcp* | F | CCTTTGGTCAGGACTCCTTC |
|  | R | AAATCAGCCAGAGCCTGTTC |
| *Psca* | F | CCACCTACTTAGCCCTGCAT |
|  | R | TGTTCATCTGTGCTGTGCAT |
| *Psmd8* | F | GTGCAAAGACATTCCCTCCT |
|  | R | GCAGGAAGAGGAGATTGAGG |
| *Ptgs2* | F | AGCCTTCTCCAACCTCTCCT |
|  | R | CAGGGATGAACTCTCTCCGT |
| *Riok1* | F | TTCGACTTCGTCACAGATCC |
|  | R | TCCTCTGGGATGCTATTTCC |
| *Rnase4* | F | GGAGCCTCTAGCTTCACACC |
|  | R | AGAGCAGAAGCAAGGACTGAG |
| *Slc25a17* | F | GGGTTTGTAGCAGGAGTGGT |
|  | R | CTGCAGCTTCAGTCTGGTGT |
| *Slpi* | F | CAAGTGCGTGAATCCTGTTC |
|  | R | GCACTTGTATTTGCCGTCAC |
| *Tmem14c* | F | AGCTACATCTGGGACCTTGG |
|  | R | GCTGTTACGGATGAGGTGAA |
| *Tpmt* | F | GAAATTGCTGGAGCCAAAGT |
|  | R | TGCTCCTCTATCCCAAATCC |
| *Zfp277* | F | GAAGCACATGATTATCGAGCA |
|  | R | AATCAGTGATGGGCTGTTCA |
| *Chrm3*  F TGGTGTGTTCTTCCTTGGAC  R ACCCAGGAAGAGCTGATGTT  **Human Genes Primer Sequence (5’-3’)** | | |
|  | | |

*B2M*  F GAGGCTATCCAGCGTACTCC

R ATGGATGAAACCCAGACACA

*CHRM3*  F TGGTTTGATGCTCCTACCTG

R AGGACAGAGGAGTGGACCAG

*ZNF277* F ACAGCAGCAAGAACGAAATG

R TGGCAATCCAATGTTGAAAG
